# Lujiatun Psittacosaurids: Understanding Individual and Taphonomic Variation Using 3D Geometric Morphometrics

**DOI:** 10.1371/journal.pone.0069265

**Published:** 2013-08-09

**Authors:** Brandon P. Hedrick, Peter Dodson

**Affiliations:** 1 Department of Earth and Environmental Science, School of Arts and Sciences, University of Pennsylvania, Philadelphia, Pennsylvania, United States of America; 2 School of Veterinary Medicine, Department of Animal Biology, University of Pennsylvania, Philadelphia, Pennsylvania, United States of America; Monash University, Australia

## Abstract

*Psittacosaurus* is one of the most abundant and speciose genera in the Dinosauria, with fifteen named species. The genus is geographically and temporally widespread with large sample sizes of several of the nominal species allowing detailed analysis of intra- and interspecific variation. We present a reanalysis of three separate, coeval species within the Psittacosauridae; *P. lujiatunensis*, *P. major*, and *Hongshanosaurus houi* from the Lujiatun beds of the Yixian Formation, northeastern China, using three-dimensional geometric morphometrics on a sample set of thirty skulls in combination with a reevaluation of the proposed character states for each species. Using these complementary methods, we show that individual and taphonomic variation are the joint causes of a large range of variation among the skulls when they are plotted in a morphospace. Our results demonstrate that there is only one species of *Psittacosaurus* within the Lujiatun beds and that the three nominal species represent different taphomorphotypes of *P. lujiatunensis*. The wide range of geometric morphometric variation in a single species of *Psittacosaurus* implies that the range of variation found in other dinosaurian groups may also be related to taphonomic distortion rather than interspecific variation. As the morphospace is driven primarily by variation resulting from taphonomic distortion, this study demonstrates that the geometric morphometric approach can only be used with great caution to delineate interspecific variation in *Psittacosaurus* and likely other dinosaur groups without a complementary evaluation of character states. This study presents the first application of 3D geometric morphometrics to the dinosaurian morphospace and the first attempt to quantify taphonomic variation in dinosaur skulls.

## Introduction

*Psittacosaurus* was first described from a well-preserved skeleton found during the Third Asiatic Expedition to Mongolia in 1922 [1], [2]. Since then, 15 species and a genus separate from *Psittacosaurus* have been placed in the Psittacosauridae [3]. The most recent review of the group accepts one genus (*Psittacosaurus*) and nine species as valid categorizing the remaining species as either junior synonyms or as nomina dubia [3]. *Psittacosaurus* is one of the most common dinosaurs currently known and is found throughout Asia (Russia, China, Mongolia, and possibly Thailand). Further, it has been inferred to have a long temporal duration from the Hauterivian to the Albian stages of the Early Cretaceous encompassing 20 Ma [4]. This wide geographical distribution in combination with a long temporal duration coupled with small body size makes the Psittacosauridae one of the most likely groups in the Dinosauria to have multiple congeneric species [5].

The purported Hauterivian dates are based on ashes from the Lujiatun beds of the Yixian Formation in Liaoning, northeastern China, which is the oldest unit producing psittacosaur skeletons [4], [6]. ^40^Ar/^39^Ar dating of ash from the Lujiatun beds interbedded with the fossiliferous layers shows that the Lujiatun specimens are Barremian (123.2±1.0 Ma) in age [7] rather than Hauterivian (128±0.2 Ma) as was earlier reported [8] and thus that the Psittacosauridae occupies a shorter temporal duration than previously supposed. The Lujiatun beds are famous for producing beautiful specimens of feathered dinosaurs and early birds (see [9] for a review), but have also produced two named species of *Psittacosaurus* (*P. lujiatunensis* and *P. major*) [6], [10] and a separate genus within the Psittacosauridae, *Hongshanosaurus houi* [11]. Sereno [3] found *Hongshanosaurus* to be a taphonomically distorted *Psittacosaurus* skull and a junior synonym of *Psittacosaurus* with *P. houi*, a nomen dubium. He found both *P. major* and *P. lujiatunensis* to be valid, though he points out many similarities between the two taxa and suggested more work needs to be done to clarify their relationships. Erickson *et al*. [12] proposed that *P. major* is synonymous with *P. lujiatunensis* reasoning that two similar species without trophic specializations would not inhabit the same environment. However, numerous extant environments in which similar species and subspecies live within the same habitats (*Buteo*, *Falco*, *Branta*, *Anolis*, *Odocoileus*) do not support this assertion [13], [14], [15].

Geometric morphometrics is an important method for demonstrating shape variation within a species given a large enough specimen sample size [16]. In one of the first studies examining species validity in dinosaurs using traditional morphometrics, Dodson [17] examined the skull of *Corythosaurus*, which resulted in reducing the number of *Corythosaurus* species from six to one. The species grouped into two separate groups, which were interpreted as two sexual dimorphs of one species [17]. Recent work using high-resolution stratigraphy of the Dinosaur Park Formation has shown that each of these supposed sexual dimorphs occupied separate stratigraphic levels and are most parsimoniously interpreted as two separate species [18]. However, the Dodson [17] study presented a valuable method for understanding species validity in dinosaurs based on traditional morphometric techniques, as well as the shortcomings of such techniques. These techniques are best applied to very closely related species, which have similar cranial proportions. Depending on the separation of proportions among species, groups may be interpreted as either separate species or the same species within the context of individual variation [17], [19].

More recently, Campione and Evans [20] examined edmontosaurs using two-dimensional geometric morphometrics to assess the validity of species of *Edmontosaurus*, *E. regalis* and *E. annectens*, along with *Anatotitan copei*, *Thespesius edmontoni*, and *Edmontosaurus saskatchewanensis*. They were able to determine that there are only two distinct cranial morphotypes in North American edmontosaurs, *E. regalis* and *E. annectens*. Such studies have also been performed on a wide range of groups, from *Protoceratops* [19], to *Podarcis* lizards [21], to mammalian carnivores [22] to examine sexual dimorphism and interspecific variation. This technique has been used in Dinosauria to examine variation and disparity between groups in sauropods [23], [24], theropods [25]–[28], ceratopsians [29], [30], hadrosaurs [20], [31], and pachycephalosaurs [32].

Studies in dinosaur cranial and appendicular morphometrics are gaining prominence in dinosaur paleontology, but three-dimensional geometric morphometric tests have not yet been performed on this group. Three-dimensional techniques are critical when examining complex objects such as skulls, which vary greatly in depth between landmarks [33]. In spite of the usefulness of morphometrics, it is not possible to replace qualitative cladistic characters with morphometric-based characters. Using cranial morphometrics combined with a reanalysis of character states has the greatest potential for unraveling the factors delineating different species of *Psittacosaurus*. We present here the first examination of *Psittacosaurus* using this approach focusing on the Lujiatun bed psittacosaurs to determine the validity of *P. lujiatunensis*, *P. major*, and *Hongshanosaurus* and quantitatively assess the range of individual and taphonomic variation within the genus *Psittacosaurus*.

## Materials and Methods

We obtained permission to visit and examine specimens in collections from all museums cited in the paper (Institute of Vertebrate Paleontology and Paleoanthropology; Dalian Museum of Natural History; Zhejiang Museum of Natural History, University of Chicago, Chinese Academy of Geological Sciences). All specimens were purchased or donated to their respective collections.

### (a) Materials

All psittacosaurid skulls used in this analysis are from the Lujiatun beds of the Yixian Formation found near Lujiatun Village, Liaoning, northeastern China in order to eliminate temporal and geographic variation. Thirty psittacosaurid skulls were digitized including the adult paratype of *Hongshanosaurus houi* (IVPP [Institute of Vertebrate Paleontology and Paleoanthropology, Beijing, China] V12617), and the holotypes *P. lujiatunensis* (ZMNH [Zhejiang Museum of Natural History, Hangzhou, China] M8137), and *P. major* (LHPV1 [Long Hao Institute for Stratigraphic Paleontology, Hohhot, Nei Mongol Autonomous Region, China]). Unfortunately the holotype of *Hongshanosaurus houi* (IVPP V12704), a juvenile specimen measuring 37.5 mm in total skull length, could not be located. A cast of the specimen was digitized instead so as to include the important holotype specimen. A number of published Lujiatun psittacosaurs were not available for study as they are currently under restudy (PKUVP [School of Earth and Space Sciences, Peking University, Beijing China] 1053, 1054, IVPP V14341) [6], [34] or were behind glass and could not be digitized (see [12], table 1 for the LPM [Liaoning Paleontological Museum, Shenyang Normal University, Shenyang, China] specimen list currently on display). Of the thirty skulls, two (DMNH [Dalian Museum of Natural History, Dalian, Liaoning, China] D2584, DMNH D1883) had to be excluded from the principal components analysis since they were missing numerous landmarks used in the analysis. These landmarks were missing due to taphonomic breakage prior to burial. Specimens displaying taphonomic variation that did not suffer breakage were included in the study so as to construct a taphonomic morphospace.

**Table 1:** A list of the 56 landmarks collected with descriptions.

Landmark Number	Description
1	Ventral tip of the rostral on midline
2	Dorsalmost part of rostralmost nasal on midline
3	Height of right nasal on skull roof
4	Rostral position of right naris (middle)
5	Dorsal position of right naris (middle)
6	Caudal position of right naris (middle)
7	Ventral position of right naris (middle)
8	Rostral position of right orbit (middle)
9	Dorsal position of right orbit (middle)
10	Caudal position of right orbit (middle)
11	Ventral position of right orbit (middle)
12	Height of right postorbital
13	Rostral position of right lateral temporal fenestra (middle)
14	Dorsal position of right lateral temporal fenestra (middle)
15	Caudal position of right lateral temporal fenestra (middle)
16	Ventral position of right lateral temporal fenestra (middle)
19	Postorbital eminence (right)
20	Dorsal position of right quadrate
22	Middle of right quadrate on caudal aspect
23	Height of right side of skull on caudalmost point (squamosal)
24	Lateralmost point of right jugal horn
25	Rostral position of right supratemporal fenestra (middle)
26	Medial position of right supratemporal fenestra (middle)
27	Caudal position of right supratemporal fenestra (middle)
28	Lateral position of right supratemporal fenestra (middle)
29	Sagittal crest at middle of supratemporal fenestrae
30	Frontal suture at middle of orbit
31	Height of left nasal on skull roof
32	Rostral position of left naris (middle)
33	Dorsal position of left naris (middle)
34	Caudal position of left naris (middle)
35	Ventral position of left naris (middle)
37	Dorsal position of left orbit (middle)
38	Caudal position of left orbit (middle)
40	Height of left postorbital
41	Rostral position of left lateral temporal fenestra (middle)
42	Dorsal position of left lateral temporal fenestra (middle)
47	Postorbital eminence (left)
51	Height of left side of skull on caudalmost point (squamosal)
52	Lateralmost point of left jugal horn
53	Rostral position of left supratemporal fenestra (middle)
54	Medial position of left supratemporal fenestra (middle)
55	Caudal position of left supratemporal fenestra (middle)
56	Lateral position of left supratemporal fenestra (middle)

Landmarks 17, 18, 21, 36, 39, 43, 44, 45, 46, 48, 49, and 50 were not used in the PCA. Landmarks 8, 11, 15, 16, 20, and 22 were not reflected onto the left side of the skull since they were missing in specimens DMNH D2156 and DMNH D1882.

With the exception of IVPP V12704, DMNH D3075-1, and DMNH D3075-3, all skulls examined in this study ranged in total skull length (back of parietal to front of rostrum) from 82 mm- 205 mm. This ranges in ages 3.5–10 years of age based on the growth curve developed for *P. lujiatunensis* by Erickson *et al*. [12]. All three juvenile skulls were found to occupy a slightly different position in the morphospace than the adult skulls. Therefore, we analyzed the morphospace for trends in allometry.

### (b) Taxonomic Methods

Morphometric techniques are not useful in directly determining taxonomic relationships due to variation from a large number of shape-based factors including sexual dimorphism, intraspecific variation, geographic variation [33], and as we demonstrate in this study, taphonomic variation. Therefore, a reanalysis of the proposed apomorphies of each species (*P. lujiatunensis*, *P. major*, and *Hongshanosaurus houi*) was performed by which each species was shown to be synonymous before morphometric analyses could be performed. Therefore, all known specimens referred to a specific Lujiatun species (IVPP V12617, IVPP V12704, ZMNH M8127, ZMNH M8138, CAGS [Chinese Academy of Geological Sciences, Beijing, China] VD04, CAGS VD05, LHPV1) were analyzed firsthand by B.P.H. (MS in preparation). Seventy-four additional specimens of *Psittacosaurus* in various degrees of preservation and ontogeny were examined including the holotypes of *P. xinjiangensis*, *P. meileyingensis*, *P. mongoliensis*, *P. gobiensis*, *P. ordosensis*, *P. sinensis*, *P. mazhongshanensis*, and *P. neimongoliensis* [1], [35]–[40]. The majority of the examined skulls were also from the Yixian Formation (n = 64), the rest of which comprised of holotype or paratype specimens from other localities. Based on the large sample size of specimens examined, it was possible to determine the wide range of individual variation present in all species level apomorphies that have been proposed to separate Lujiatun psittacosaurids.

### (c) Morphometric-based Methods

Three-dimensional data were collected using a Polhemus FastSCAN 3D surface scanner and stylus. Taking landmarks directly using a stylus or indirectly from a digitized scan has both advantages and disadvantages. Stylus-derived landmarks are more accurate than scan-derived landmarks because it is possible to manipulate the actual specimen when they are collected so that the landmark can be taken precisely, though this can be tedious in very small skulls. Scan-derived landmarks have the advantage that they are more easily repeatable in follow-up studies than stylus-derived landmarks. Stylus-derived landmarks are recorded as numbers in a datasheet and are much more difficult to visualize than scan-derived landmarks, which appear onscreen in their original orientation. However, wireframes created in programs such as *morphologika* ^2^ [41] can aid in visualization of stylus-derived landmarks. Landmarks used in statistical analyses in this study are all stylus-derived, but scans of each examined skull were also taken for reference.

The scanner allows for manual rotation of each skull via a second receiver attached to the base of each skull. Many examined skulls had matrix in their interiors so that attaching the receiver directly on the skull was not necessary and the bone itself was not compromised. For skulls that had been fully prepared, the receiver was attached to the braincase. As this region is frequently obscured by matrix, it was not necessary to have a high-resolution scan of the braincase making this the optimal region for receiver placement. Each scan was collected three times in order to ensure an accurate capture of skull shape. Scans are available upon request (a scan of ZMNH M8137 is included in Multimedia S1).

**Figure 1:**
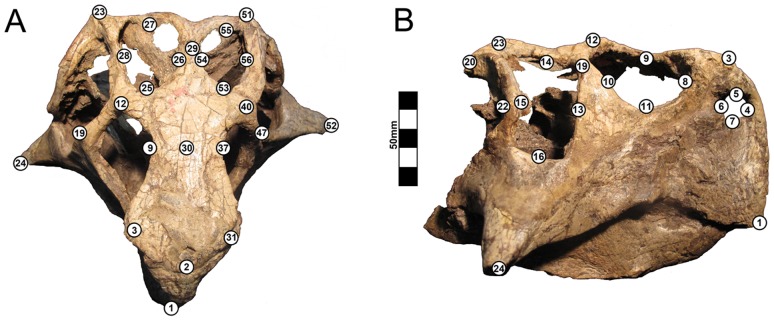
Landmark locations. The locations of the 3D landmarks are presented here in (A) dorsal and (B) lateral views on ZMNH M8137. Since the landmarks were not reflected on either side of the skull, the left lateral landmarks have different landmark numbers than the right lateral landmarks. A 3D model of the skull of ZMNH M8137 is included in Multimedia S1 for reference. Scale = 50 mm.

Fifty-six landmarks were collected using the mechanical stylus attachment on the Polhemus FastSCAN system (Figure 1; Table 1). Landmarks 17, 18, 21, 36, 39, 43, 44, 45, 46, 48, 49, and 50 were excluded from the final analysis since they were missing in several of the twenty-eight specimens. It was deemed more desirable to have a higher sample size of specimens than to have a higher sample size of landmarks considering that forty-four landmarks were still available for analysis after these were eliminated. The large number of landmarks was necessary due to the inability to reflect the right and left sides into a single landmark set. This is because taphonomic variation of the skull differs on the right and left sides due to differential compression and this is a major focus of the present analysis.

Once the landmarks were collected, they were analyzed in *morphologika* ^2^ [41]. Specimens were rescaled and rotated using Generalized Procrustes Analysis (GPA) in order to adjust for size and eliminate it as a contributing factor so as to establish only shape differences [33]. A GPA minimizes the sums of squared distances between the landmarks in each specimen by centering them on a common centroid by rotating and rescaling the landmark configurations [42]. The data are then put into a principal components analysis in order to partition variance allowing us to visualize changes in shape irrespective of size. Principal components analysis (PCA) is a critical method when dealing with large numbers of landmarks in three-dimensions, as there are three times the number of landmark coordinates in a plane in three dimensions as there are total number of landmarks. PCA reduces the dimensionality of large multivariate datasets by creating linear combinations of the original data so that it can be more easily analyzed. The broken stick method was used to determine the number of principal components that had biologic meaning [33]. Confidence ellipses at the 95% confidence interval were created in MATLAB (2010, The Mathworks, Natick, MA) using the formula, x¯ - 1.96σ≤μ ≥ x¯ +1.96σ, for each principal component. Details on 2D and 3D morphometrics and statistical analyses can be found in Bookstein et al. [43] Bookstein [44], Rohlf and Bookstein [45], and Zelditch *et al*. [33].

Specimens were all examined within a two-month interval and all landmarks were taken by B.P.H. to minimize intra-observer error and remove inter-observer error. Further, landmarks were taken on the holotype of *P. major*, (LHPV1) ten separate times in order to create an error sample. Euclidean distances for PC1, PC2, PC3, and PC4 were calculated by subtracting the mean of the error sample from each PC coordinate for the remaining 27 specimens in order to determine any overlap between the LHPV1 error sample and the other specimens. Euclidean distances between specimens are included in the Table S2 in File S1. Methods follow Lockwood *et al.* [46].

Unfortunately the limitation of this dataset is that each species has only two skulls that have previously been referred to a specific Lujiatun species, with the rest being *Psittacosaurus* sp. Less exploratory analyses such as canonical variates analysis or discriminate function analysis were not performed on the data due to the lack of any visible groupings in the PCA partially resulting from the small sample size of each nominal species. In order to assess changes in the taphomorphospace among the three species, all three species and 23 previously unstudied psittacosaur skulls from the Lujiatun beds were plotting using PCA. By establishing confidence ellipses around the data, it is possible to determine which specimens are statistically separate from the mean of all of the included specimens. Critically it should be noted that specimens outside of the confidence ellipse are not statistically different from any other group, but they are statistically different from the mean of all examined samples.

## Results

### (a) Taxonomic-based results

In order to demonstrate whether or not the character states of the three Lujiatun psittacosaur species are appreciably different, it was first necessary to review the apomorphies of each species on the bases of which they were originally erected. Therefore, the taxa are reviewed here and the significance of the autapomorphies and character combinations are analyzed. Apomorphies in the most recent comprehensive review of *Psittacosaurus* [3] as well as each species' original description were assessed. Following the review of characters we evaluate the validity of each character in separating out any particular Lujiatun psittacosaurid species from any other species. Characters and their distribution in each species are summarized in Table 2.

**Table 2:** List of characters used to describe each Lujiatun psittacosaurid species.

Characters	Citation	Character suggested for:	1	2	3	4	5
Prefrontal width less than 50% the width of the nasal	Sereno, 2010; Zhou *et al*., 2006	1	√	√		√	
Quadratojugal-squamosal contact along the anterior margin of the quadrate	Sereno, 2010; Zhou *et al*., 2006	1	√			√	√
Jugal-quadrate contact posteroventral to the laterotemporal fenestra	Sereno, 2010	1	√			√	√
Upturned maxillary protuberance	Zhou *et al*., 2006	1	√				
Jugal horn that arises in the posterior portion of the skull and projects posterolaterally	Zhou *et al*., 2006	1	√			√	√
Closed external mandibular fenestra	Zhou *et al*., 2006	1	√	√	√		
Large angular	Zhou *et al*., 2006	1	√	√		√	
Depression in the center of the anterior surface of the jugal	Zhou *et al*., 2006	1	√	√		√	
Rounded ridge extending halfway along the orbital ramus of the jugal	Zhou *et al*., 2006	1	√	√		√	
Primary ridge on the teeth with an enlarged central lobe	Zhou *et al*., 2006	1	√	√	√		
Maximum width across the nasals and interorbital frontal width is subequal to the width of the rostral	Sereno, 2010	2	√	√		√	√
Tall subtriangular lateral femoral fenestra with anteroposterior width of the ventral margin 25% the dorsoventral height	Sereno, 2010	2	√	√		√	√
Anterior ramus of jugal is convex	Sereno, 2010	2	√	√		√	
Elongate basipterygoid	Sereno, 2010	2		√			
Hypertrophied dentary flange with the anterior corner 30% of the depth of the dentary ramus	Sereno *et al*., 2007; Sereno, 2010	2	√	√		√	
Seven sacral vertebrae	Sereno *et al*., 2007; Sereno, 2010	2					
Skull much longer relative to the body than other species	Sereno *et al*., 2007	2					
Transversely narrow dorsal skull roof	Sereno *et al*., 2007	2		√		√	√
Ventrolaterally projecting jugal horn	Sereno *et al*., 2007	2		√		√	√
Absence of the external mandibular fenestra	Sereno *et al*., 2007	2	√	√	√		
Prominent jugal-quadratojugal process below the maxillary tooth row	You *et al*., 2003	3			√		√
Long preorbital region	You and Xu, 2005	3			√		√
Elliptical and caudodorsally oriented orbit	You *et al*., 2003	3			√		√
Lateral temporal fenestra with its major axis oriented caudodorsally	You and Xu, 2005	3			√		√

A number of characters are seen in multiple species, are taphonomically variable and are not true characters, or are seen gradationally across a large sample set of Lujiatun psittacosaurids suggesting that the absolute seen in any particular specimen is not a character separating species, but is an extreme on the end of a gradual continuum. 1 = *P. lujiatunensis*, 2 = *P. major*, 3 = *Hongshanosaurus*, 4 = Characters gradationally variable among 25 Lujiaun *P.* sp studied, 5 = Taphonomically variable characters.

#### (i) Psittacosaurus lujiatunensis - ZMNH M8137 (holotype), M8138 (paratype)

In the most recent review of *Psittacosaurus* taxonomy, *P. lujiatunensis* was considered valid with three distinct autapomorphies [3]. *P. lujiatunensis* has (1) a prefrontal width less than 50% of the width of the nasal, (2) quadratojugal-squamosal contact along the anterior margin of the quadrate, and (3) a jugal-quadrate contact caudoventral to the lateral temporal fenestra [3]. In the original description, Zhou *et al*. [6] describe the autapomorphies as (1) narrow prefrontals that are less than 50% of the width of the nasal, (2) an upturned maxillary protuberance, (3) a jugal horn that arises in the caudal portion of the skull and projects caudolaterally, (4) the ventral ramus of the squamosal contacts the quadratojugal, (5) a closed external mandibular fenestra, (6) a large angular, (7) a depression in the center of the rostral surface of the jugal, (8) a rounded ridge extending dorsally across the maxilla-jugal suture that ends halfway along the orbital ramus of the jugal, and (9) a primary ridge on the teeth with an enlarged central lobe.

Additional synapomorphies described by Zhou *et al*. [6] are also examined here in order to better understand *P. lujiatunensis*. As in *P. sinensis* and some specimens of *P. mongoliensis*, the skull is wider than it is long. There is a low ridge on the surface of the premaxilla, which is also seen in *P. mongoliensis*, *P. meileyingensis*, and *P. major* [6], [37], [47]. There is a deep antorbital fossa as in other psittacosaur species [6]. There is a weak postorbital prominence also seen in *P. meileyingensis* [6] and *P. major*. The quadrate shaft is strongly concave caudally as in *P. sinensis* and *P. meileyingensis* [6] and has been noted by the author in numerous Lujiatun psittacosaurid skulls that are undistorted. There is no caudal process on the pterygoid [6]. As in many other psittacosaur species, there is also a prominent dentary flange [6].

Prefrontal width less than 50% of the nasal width is a character seen in *P. lujiatunensis*, but also to varying degrees in the sample set of 25 Lujiatun *Psittacosaurus* sp. specimens. *P. major* has very wide prefrontals, but within the range of individual variation based on the 25-sample subset. Therefore, this character is interpreted as individually variable within the Lujiatun psittacosaur species rather than as an autapomorphy of *P. lujiatunensis*. The quadratojugal-squamosal contact along the rostral aspect of the quadrate is only seen in ZMNH M8137. However, the ventral ramus of the squamosal and the dorsal ramus of the quadratojugal are almost always broken in Lujiatun psittacosaur specimens as in LHPV1 (holotype of *P. major*) and IVPP V12617 (adult paratype of *Hongshanosaurus*) so this character cannot be effectively evaluated in either of these taxa. The jugal-quadrate contact is noted as caudoventral to the lateral temporal fenestra in *P. lujiatunensis* [3]. However, the contact is just dorsal to the ventral aspect of the lateral temporal fenestra. This contact is about 30% above the ventral margin of the lateral temporal fenestra in *P. major*, but due to distortion this feature is likely a taphonomic artifact. The location of the contact is widely variable in the sample of *Psittacosaurus* sp. and does not cluster into two distinct groups (just dorsal to the ventral lateral temporal fenestra and 30% above the ventral margin of the lateral temporal fenestra) as would be expected in two separate species.

Zhou *et al*. [6] notes the maxillary protuberance as upturned, but this feature is identical to that of other psittacosaurs possessing a large maxillary protuberance and could be an allometric feature due to the large size of ZMNH M8137. The direction of the jugal horn is widely variable among psittacosaur species and is due to taphonomic distortion of the skull. As the jugal horns are relatively thin and project outward, they are skull element most susceptible to compression. A closed external mandibular fossa is a feature shared by all three Lujiatun psittacosaur species, though this region is broken in some specimens. The large angular in *P. lujiatunensis* is an ontogenetically variable character and is also seen in *P. major* (LHPV1), another large specimen. The depression in the center of the rostral surface of the jugal is seen in both *P. lujiatunensis* and *P. major*. The rounded ridge extending along the jugal noted in *P. lujiatunensis* [6] continues into the maxilla and is synonymous with the convex rostral ramus of the jugal, which is a character of *P. major* [3].

#### (ii) Psittacosaurus major – LHPV1 (holotype), CAGS VD04 (referred)

*P. major* as the name suggests, is based on a large *Psittacosaurus* skull and associated postcranial material. The skull is suggested to be similar to *P. mongoliensis* except that it is 25% larger in comparison with its associated postcranium than *P. mongoliensis* skulls and postcrania [10]. Sereno [3] names six autapomorphies for *P. major*: (1) the maximum width across the nasals and interorbital frontal width is subequal to the width of the rostral, (2) tall, subtriangular lateral temporal fenestra with rostrocaudal width of the ventral margin approximately 25% of the dorsoventral height, (3) the rostral ramus of the jugal convex, (4) elongate basipterygoids as measured from the notch between the processes to the basal tubera, (5) hypertrophied dentary flange with the rostral corner approximately 30% of the depth of the dentary ramus and with only a short gap to the predentary, and (6) possession of seven sacral vertebrae. The highlighted differences between *P. lujiatunensis* and *P. major* are the elongation of the basipterygoid in *P. major* and some differences in sutural contacts [3].

The original description of *P. major* notes (1) a skull much longer relative to its body than other *Psittacosaurus* species, (2) a transversely narrow dorsal skull roof, (3) accentuated dentary flange with a depth approximately one third that of the mandibular ramus, (4) a ventrolaterally projecting jugal horn, (5) absence of the external mandibular fenestra (as in *P. sinensis*, *P. neimongoliensis*, and *P. lujiatunensis*), (6) and seven sacral vertebrae as opposed to six as in all other species of *Psittacosaurus* [10]. A follow-up paper describing a completely prepared Lujiatun *Psittacosaurus* skull (CAGS VD04) refers the specimen to *P. major* based on its transversely narrow skull roof and very prominent dentary flange [5]. They further list many more cranial features. The specimen shows an elliptical median interpremaxillary foramen (seen in *Hongshanosaurus* and *P. lujiatunensis*), prominent neurovascular canals on the internal wall of the beak, long divergent basipterygoid processes developed as vertical blades with a deep cleft dividing them, and a horizontally oriented vomer bone [5].

Other apomorphies include prominent jugal horns, large nares, laterally flaring palpebrals (as opposed to caudally flaring palpebrals in *P. lujiatunensis*), and lateral temporal fenestrae that are narrower ventrally than they are dorsally (as in *P. lujiatunensis*) [5]. The nasals are narrow and squeezed between the prefrontals, unlike *P. lujiatunensis* [5]. However, this character does not seem to be highly divergent between *P. major* and *P. lujiatunensis* considering a large range of variation in the prefrontal region in the 25-specimen subset. The frontals are reconstructed in *P. lujiatunensis* as sharing a triangular rostral border with the nasals [6]. They are reconstructed as having a flat border in *P. major*, although the rostral and caudal borders of the frontals are said to be difficult to determine due to blurring of suture lines [5]. The large skull size noted by Sereno *et al*. [10] is regarded as an unreliable character by Sereno [3] and relative large skull size is a trait shared by *P. major*, *P. lujiatunensis*, and *P. sinensis*. The dentary flange has a prominence that extends from the coronoid process to the rostral border of the flange [5]. The flange itself is large and ventrolaterally projecting [5], [10]. The flange is caudally placed on the ramus as in *P. lujiatunensis* [5].

The relative nasal width, interorbital width, and the rostral width are all subequal in *P. lujiatunensis*, *P. major*, and many of the specimens of *Psittacosaurus* sp. The tall, subtriangular lateral temporal fenestrae in *P. major* are also seen on the holotype skull of *P. lujiatunensis* (ZMNH M8137). The size and shape of the lateral temporal fenestra varies widely on ZMNH M8137 from a tall, narrow fenestra on the right side to a short, wide fenestra on the left side. This demonstrates that this character is highly taphonomically variable [3]. The extreme hypertrophy of the dentary flange in *P. major* is also considered an autapomorphy [10]. However, both the holotype and paratype of *P. lujiatunensis* have hypertrophied dentary flanges, demonstrating that this character simply develops in late ontogenetic stages as it is only seen in the largest specimens. The transversely narrow skull roof seen in *P. major* is taphonomically variable and is variable among the *Psittacosaurus* sp. subset. As with *P. lujiatunensis*, the ventrolaterally projecting jugal horn of *P. major* is widely variable and is a result of skull compression.

*P. major* was inferred by Sereno *et al*. [10] to have a large skull relative to its body size. The skull-femur ratio ranges from 0.85–1.38 in a dataset of 43 psittacosaurs. The holotype of *P. lujiatunensis* has a skull-femur ratio of 0.99 and LHPV1 has a ratio of 1.13 so both are within the range of values for a large dataset of psittacosaurs, showing that neither has an anomalously large skull relative to body size (Table S3 in File S1). Additionally, Sereno *et al*. [10] characterize *P. major* as having seven sacral vertebrae. However, the cranialmost vertebra does not contact the ilium on either side and cannot be confirmed as a sacral. Articulation points on the medial ilium could potentially be broken contacts for sacral ribs, but the assertion that *P. major* only has six sacral vertebrae is supported by the fact that all other examined psittacosaurids from the Lujiatun sample have six sacral vertebrae. Therefore it is most likely that *P. major* also has six sacral vertebrae.

#### (iii) Hongshanosaurus houi – IVPP V12704 (holotype), IVPP V12617 (paratype)

The second nominal genus within the Psittacosauridae from the Lujiatun beds is *Hongshanosaurus*, with a single species, *H. houi* [11]. The genus was erected on the basis a complete juvenile skull with no postcranial material [11], although an adult skull has since been referred to the taxon [48].

*Hongshanosaurus* is distinguished from *Psittacosaurus* by (1) a prominent jugal-quadratojugal process below the maxillary tooth row, (2) a long preorbital region, and (3) an elliptical and caudodorsally oriented orbit [11]. In addition, it does not have an antorbital fenestra (as in all species of *Psittacosaurus*) and has very long nasals [11]. It is placed in the Psittacosauridae based on the caudodorsal process of the premaxilla, contact between the premaxilla and lacrimal, long rostral process on the nasal, open canal on the lateral surface of the lacrimal, and having fewer than ten maxillary teeth in either ramus [11]. The adult specimen is referred to *Hongshanosaurus* on the basis of the preorbital region being half of the total skull length, elliptical nares and orbits, and lateral temporal fenestrae with their major axis oriented caudodorsally [46]. It also has laterally flaring jugal horns and a large dentary flange, just as in *P. major* and *P. lujiatunensis*. You and Xu [46] view these as ontogenetic characters in *Hongshanosaurus*.

The type material for *Hongshanosaurus houi* has previously been considered dorsoventrally flattened [3], but the adult material has been suggested to be undistorted based on completely undeformed palatal features [48]. However, there is a significant amount of plaster connecting the palate with the braincase in IVPP V12617 suggesting that the skull was generally compressed, but that palate was not distorted (Figure S4 in File S1). This is supported by the fact that all of the apomorphies distinguishing *Hongshanosaurus* can be explained via dorsoventral crushing. The long preorbital region, elliptical, caudodorsally oriented orbit and lateral temporal fenestra, and the jugal-quadratojugal process located ventral to the maxillary tooth row all would occur if the entire skull were taphonomically distorted such that the caudal aspect of the skull is dorsoventrally compressed and rotated about the undistorted rostral aspect of the skull. Varying degrees of these features are seen in Lujiatun *Psittacosaurus* sp. specimens based on their degree of dorsoventral compression.

### (b) Morphometric-based results

**Figure 2:**
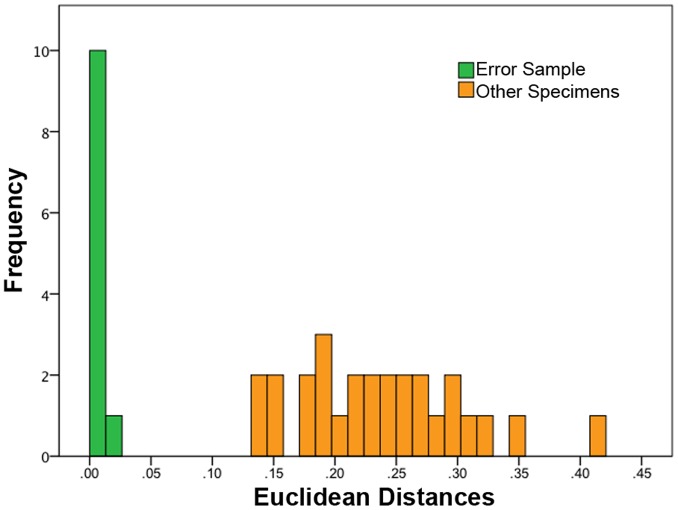
Euclidean distance error test. Euclidean distances were calculated for PC1-4 for all 28 specimens and the 10 additional error specimens (LHPV1). The error specimens all grouped together with no overlap from other specimens.

PCA was run both with juvenile specimens (DMNH D3075-1, DMNH D3075-3, and IVPP V12704) included (N = 28) and with only adult specimens (N = 25) since the juvenile skulls occupy a different part of the morphospace from the larger skulls. In general, the adult-only PCA caused the cluster to be more closely aligned with the 95% confidence ellipses (Figure S1, S2 in File S1). This did not substantially affect the grouping within the morphospace, but did change the locations of particular specimens in some instances. These two separate analyses are referred to as the 28-specimen PCA (with juveniles) and the 25-specimen PCA (adults only). Given the similarities between the two groupings, the 28-specimen PCA is presented here (see File S1 for discussion of the 25-specimen PCA). The first four principal components are interpreted. The traditional method for determining the number of principal components used in an analysis is the broken-stick method, whereby the principal components to the left of an inflection point on a scree plot are considered significant. The first two PCs had much higher eigenvalues than the remaining PCs (Table 3). The following three PCs signified a second tier of eigenvalues. The first four PCs are here examined and comprise 63.2% of the total variance. Four PCs were chosen in this analysis as they represent different aspects of taphonomic variation demonstrated by the entire sample. Successive PCs separated out single specimens or small groups of specimens and were therefore difficult to interpret and are not discussed. The Euclidean distances between the error sample and the rest of the samples had no overlap showing that there was no substantial difference in the way the landmarks were measured from specimen to specimen (Figure 2).

**Table 3:** Principal Components and Eigenvalues.

Principal Component Number	Eigenvalues	Proportion of Total Variance	Cumulative Variance
PC1	8.56E-03	27.9%	27.9%
PC2	5.71E-03	18.6%	46.5%
PC3	2.77E-03	9.04%	55.6%
PC4	2.34E-03	7.64%	63.2%
PC5	2.12E-03	6.92%	70.1%
PC6	1.67E-03	5.45%	75.6%
PC7	1.33E-03	4.35%	79.9%
PC8	8.29E-04	2.70%	82.6%

All 28 principal components and their associated eigenvalues can be found in Table S5 in File S1.

**Figure 3:**
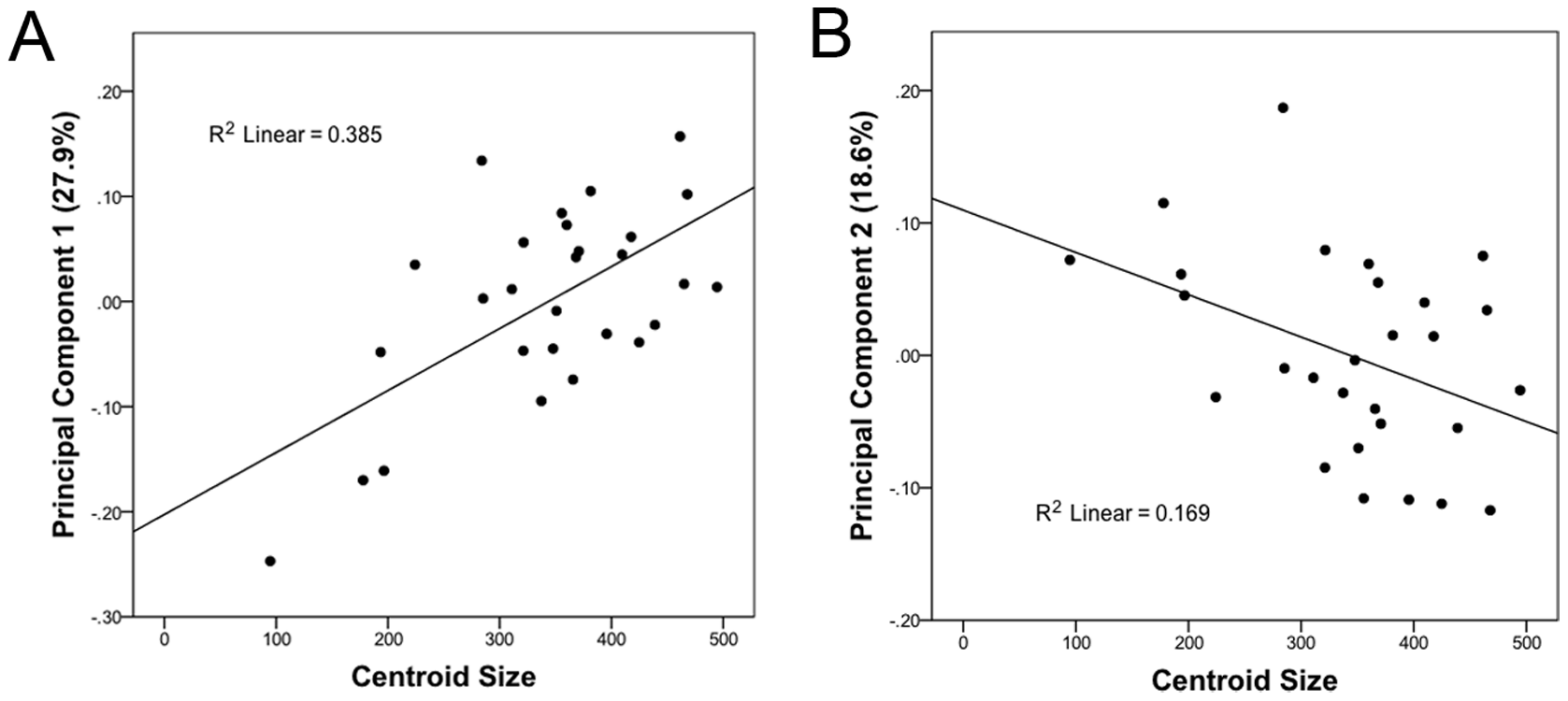
Allometric Analysis. By plotting centroid size against each principal component, it is possible to determine if there is any allometric effect on the PCA. R^2^ values are low between all PCs and centroid size.

In order to test for allometric effects, each principal component examined (PC1–PC4) was plotted against centroid size (Figure 3). Each linear fit had a low R^2^ value. The Spearman's rank correlation coefficient, r_s_, was calculated to evaluate if there was a significant correlation between principal components and centroid size. This nonparametric option was chosen because the centroid size of our sample was not normally distributed. PC3 and PC4 did not have significant correlations with centroid size so allometry did not have a significant impact on them (p = 0.392 and p = 0.272 respectively). PC1 and PC2 did have a significant correlation with centroid size (p<0.001 and p = 0.029 respectively). Removing the small specimens (IVPP V12704, DMNH 3075-1, DMNH 3075-2) from the dataset eliminated the significant allometric correlation in PC1, but PC2 and PC4 were found to be significant (Table S4 in File S1). (PC1, p = 0.756; PC2, p = 0.038; PC3, p = 0.933; PC4, p = 0.283). ZMNH M8138 is a clear outlier in the 25-specimen dataset on PC2 (Figure S1 in File S1) and if it is taken out of the dataset, PC2 is no longer significantly correlated with centroid size (p = 0.235).

It is likely that the correlation between PCs and centroid size is not driven by allometry, but is driven by differential distortion of the smaller skulls. The small size of the smallest specimens predisposes them to more substantial crushing than the larger specimens due to a lack of fusion in the skulls resulting from early ontogenetic stage. Additionally, removal of a single specimen from the analysis caused a change in the significance of the correlation for PC2 suggesting small sample size is a dominant factor in producing significant p-values here. It is not possible to eliminate the smaller specimens from the analysis because understanding the placement of the holotype of *Hongshanosaurus* (IVPP V12704) in the morphospace is one of the main goals of the study. We therefore make the assumption in this study that determination of significant correlation between PCs and centroid size is related to taphonomic factors and small sample size rather than an allometric signal.

**Figure 4:**
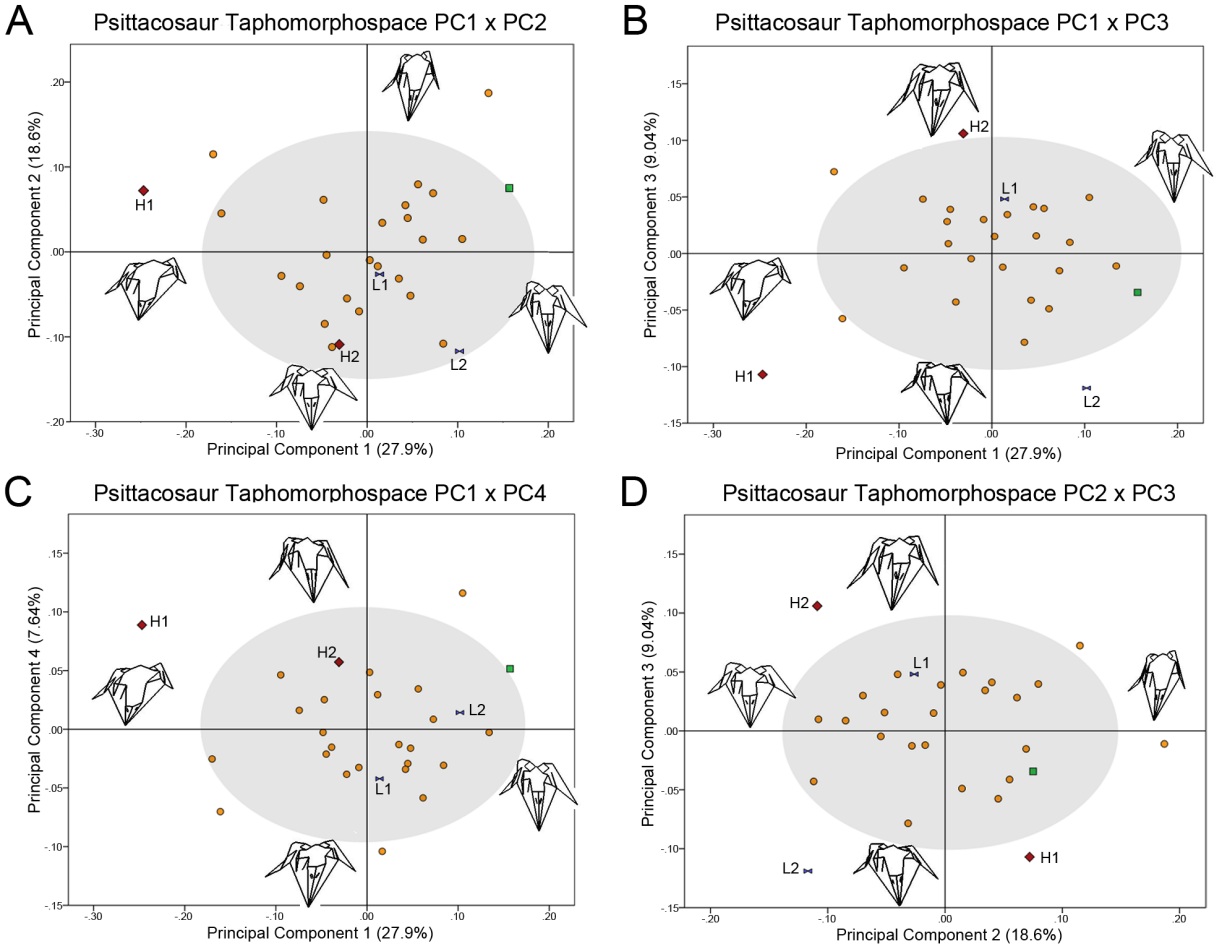
Psittacosaurid taphomorphospaces. Each taphomorphospace is generated with principal component axes. Blue bowties = *P. lujiatunensis*. Green squares = *P. major*. Red diamonds = *Hongshanosaurus*. Orange circles = *Psittacosaurus* sp. H1 = IVPP V12704; H2 = IVPP V12617; L1 = ZMNH M8137; L2 = ZMNH M8138. Gray circles represent 95% confidence ellipses of the mean of all specimens. Wireframes for each principal component axes are presented next to their respective principal component axis. Each wireframe was generated on each respective axis. (A) PC1×PC2, (B) PC1×PC3, (C) PC1×PC4, and (D) PC2×PC3.

The confidence interval for each PC is determined by the mean based on all 28 specimens and is displayed graphically in the confidence ellipse. The 95% confidence interval for PC1 is −0.181≤μ ≥0.181, PC2 is −0.148≤μ ≥0.148, PC3 is −0.103≤μ ≥0.103, and PC4 is −0.095≤μ ≥0.095. Specimen data including principal component coordinates is included in Table S1, S4 in File S1. The first principal component comprises 27.9% of the variance. A strongly positive PC1 score is characterized by a tall skull with ventrally projecting jugal horns (Figure 4A). The rostrum in lateral view is oriented at 90° with erect nasals in comparison with a flattened sloping rostrum. By contrast, a strongly negative PC1 score is characterized by a dorsoventrally flattened skull with incipient jugal horns. The rostrum is also sloping as opposed to erect. Only IVPP V12704 (holotype of *Hongshanosaurus*) is outside the 95% confidence intervals for PC1.

Principal component 2 represents 18.6% of the total variance. A strongly positive PC2 score is represented by a laterally compressed skull with ventrally projecting jugal horns and an erect rostrum (Figure 4B, 4D). A strongly negative PC2 score is characterized by a broad skull with laterally flaring jugal horns and a taller rostrum than caudal aspect of the skull. DMNH D2590 is outside of the 95% confidence interval on the positive PC2 axis. DMNH D3075-1 is outside of the 95% confidence ellipse on the negative PC2 axis. DMNH D3075-3 also groups with DMNH D3075-1, but is within the 95% confidence ellipse. DMNH D3075-1, D3075-3, and IVPP V12704 are all juveniles and occupy a slightly separate morphospace from the main sample. However, it would appear that the grouping is based on similar taphonomic distortion rather than allometry (Figure 4).

Principal component 3 is comprised of 9.04% of the total variance. The strongly positive PC3 axis is represented by a tall skull crushed inward on the right side (Figure 4B, 4D). IVPP V12617 is outside of the 95% confidence interval on the positive PC3 axis. It does not have marked asymmetry at the midline, but is dorsoventrally deformed. The strongly negative PC3 axis is composed of specimens with a dorsoventrally flattened skull that is crushed inward on the left side. IVPP V12704 and ZMNH M8138 are outside the 95% confidence interval on the negative PC3 axis. Both are dorsoventrally crushed and have differential crushing on the left side. Crushing on a single side of the skull is common in the dataset and therefore PC3 represents a valuable quantification of this feature in spite of accounting for a relatively small percent of the total variance.

Principal component 4 comprises 7.64% of the total variance. A strongly positive PC4 is represented by a caudally angled skull with ventrolaterally oriented jugal horns and a flattened rostrum (Figure 4C). This causes an exaggeration of the length of the rostrum. Both specimens of *Hongshanosaurus* group on the strongly positive PC4 axis though are within the 95% confidence interval. DMNH D2590 is the only specimen outside of the 95% confidence interval and is also crushed in such a way as it has an elongate rostrum. A strongly negative PC4 is represented by skulls with an erect rostrum that is much taller than the caudal aspect of the skull. DMNH D2592 is outside the 95% confidence interval on the negative PC4 axis.

## Discussion

*Psittacosaurus* is one of the most speciose dinosaur genera with fifteen separate nominal species [3]. *Psittacosaurus* is undoubtedly geographically widespread and is found as far north as Siberia (*P. sibiricus*; [49]), as far west as Xinjiang, China (*P. xinjiangensis*; [36]), as far south as Thailand (*P. sattayaraki*, [50]); and along the eastern coast of China (*P. sinensis*, *P. lujiatunensis*; [6], [35]). Lucas [4] further suggested a long ‘*Psittacosaurus* biochron’ of 20 million years. These factors together would imply the potential for a speciose clade given the excellent preservation of Early Cretaceous fossiliferous sediments in Asia. However, recent work has shown that the *Psittacosaurus* biochron was shorter than previously suggested [3], [7], which perhaps in turn implies a smaller likelihood of the Psittacosauridae being as speciose as previously supposed.

Based on a reanalysis of the characters used to distinguish the three Lujiatun psittacosaurs, *P. lujiatunensis*, *P. major*, and *Hongshanosaurus houi*, using all referred specimens as well as a large number of complete *Psittacosaurus* skulls hitherto undescribed also from the Lujiatun beds of the Yixian Formation, it is concluded that *P. major* and *Hongshanosaurus* are both junior synonyms of *P. lujiatunensis*. *Hongshanosaurus* You *et al*. [11] was named before *P. lujiatunensis* Zhou *et al*. [6], but we argue that *P. lujiatunensis* should be retained as the species name for the Lujiatun psittacosaurid species since the holotype of *Hongshanosaurus* (IVPP V12704) is clearly a juvenile and does not have many of the characters distinguishing this taxon from other psittacosaurids due to the early ontogenetic stage of the skull of IVPP V12704 [3].

**Systematic Paleontology:**

**Dinosauria Owen, 1842**

**Ornithischia Seeley, 1888**

**Ceratopsia Marsh, 1890**

***Psittacosaurus*** **Osborn 1923**

***Psittacosaurus lujiatunensis*** **, Zhou** ***et al*** **. 2006**

2007 *Psittacosaurus major*, Sereno *et al.*, p. 275.

2003 *Hongshanosaurus houi*, You *et al.*, p. 15.

Holotype: ZMNH M8137, skull and nearly complete postcranial skeleton. Paratypes: ZMNH M8138, PKUVP V1053, PKUVP V1054 [6], LHPV1 [10], IVPP V12617 [48]

Referred Specimens: DMNH D1882, DMNH D1883, DMNH D2581, DMNH D2582, DMNH D2583, DMNH D2584, DMNH D2585, DMNH D2586, DMNH D2587, DMNH D2588, DMNH D2589, DMNH D2590, DMNH D2591, DMNH D2593, DMNH D2594, DMNH D2595, DMNH D2598, DMNH D2599, DMNH D2600, DMNH D3419.

Type locality: Lujiatun Village, near Beipiao City, Liaoning, China; Lujiatun beds, lowest part of the Yixian Formation; Barremian, Early Cretaceous [7].

### (a) The three primary Lujiatun psittacosaurid taphomorphotypes

**Figure 5:**
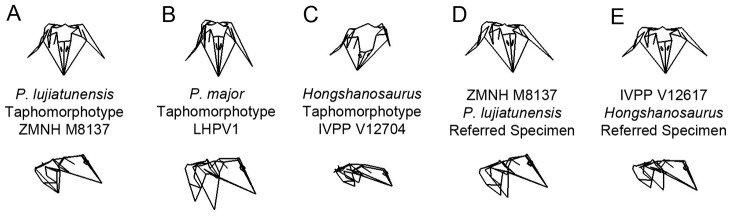
Psittacosaurid wireframes. Wireframes generated from craniometric landmarks in *morphologika^2^* showing taphonomic deformation in rostral (top) and lateral (bottom) views for (A) ZMNH M8137, (B) LHPV1, (C) IVPP V12704, (D) ZMNH M8138, and (E) IVPP V12617. These wireframes were created in the PC1×PC2 morphospace. A–C each represents a different taphomorphotype of *P. lujiatunensis*.

The morphometric grouping of Lujiatun psittacosaurid specimens into a single cluster in morphospace also supports the interpretation of all Lujiatun psittacosaurids representing a single species. The variability across the PCA cluster is largely based on taphonomic deformation of the skull (Figure 4), which can be seen in wireframe reconstructions (Figure 5). Considering that a number of the characters applied to each of the Lujiatun psittacosaurs are influenced by taphonomic deformation, we refer to each nominal Lujiatun psittacosaur species as taphomorphotypes rather than as separate biological species. Each taphomorphotype is based on the holotype specimens of its proposed species (*P. lujiatunensis* = ZMNH M8137; *P. major* = LHPV1; *Hongshanosaurus* = IVPP V12704) (Figure 5).

The *P. lujiatunensis* taphomorphotype is relatively undistorted in comparison with other *Psittacosaurus* skulls examined. This is demonstrated by its location near the consensus shape in all PC plots (Figure 4) as well as overall morphology. Therefore, the *P. lujiatunensis* taphomorphotype can be used as a baseline for examining other taxa. In contrast, the paratype ZMNH M8138 groups further from the mean due to some dorsoventral crushing confined especially to the caudal aspect of the skull. This causes it to group outside of the 95% confidence intervals for the mean of PC3, which is primarily characterized by asymmetric dorsoventral crushing. In spite of the fact that ZMNH M8137 and ZMNH M8138 are both *P. lujiatunensis* [6], they group in widely different regions of the morphospace and ZMNH M8138 is more characteristic of the *Hongshanosaurus* taphomorphotype rather than the *P. lujiatunensis* taphomorphotype (Figure 5).

The *P. major* taphomorphotype is laterally compressed giving a tall skull relative to its width (Figure 5). Both highly positive PC1 and PC2 axes represent mediolateral compression in the form of the *P. major* taphomorphotype. LHPV1 plots in the far right corner of the PC1×PC2 plot and has both a highly positive PC1 and PC2 score (Figure 4A). You *et al*. (2008) describe an additional specimen of *P. major*, CAGS VD04, which was not included in this dataset, but that also had a tall, laterally compressed skull. The attribution to *P. major* of this specimen demonstrates the importance of characterizing taxa on the basis of non-taphonomic characters. DMNH D2590 presents an even more extreme form of mediolateral compression and plots further into this region of the morphospace than the *P. major* taphomorphotype. This skull shares the compressional characters of *P. major* such as the transversely narrow skull roof and lateral temporal fenestra shape, but has no clear apomorphies distinguishing it from ZMNH M8137.

The *Hongshanosaurus* taphomorphotype is represented by a dorsoventrally compressed skull with an elongate rostrum. IVPP V12704 is outside the confidence interval for PC1 and PC3 (Figure 4B). The strongly negative PC1 and PC3 axes are both characterized by dorsoventrally crushed skulls. Many of the features caused by dorsoventral compression in this taxon were extreme enough that they were interpreted as autapomorphies of a new genus [11] demonstrating the importance of understanding taphonomic distortion in a wide range of specimens of closely related animals when possible.

The adult specimen of *Hongshanosaurus* (IVPP V12617) plots in a different region of the morphospace from IVPP V12704 in PC1, PC2, and PC3, but in the same region for PC4 (Figure 4). IVPP V12617 does not represent the *Hongshanosaurus* taphomorphotype as it is dorsoventrally crushed differently from IVPP V12704 (Figure 5). IVPP V12704 is completely dorsoventrally crushed to the same degree in all regions of the skull [3] whereas IVPP V12617 is crushed primarily in the caudal aspect of the skull exaggerating the relative size of the rostrum while the rostrum itself is not strongly compressed. The deformation of IVPP V12617 caused the presence of all of the characters of *Hongshanosaurus* [48] without causing it to plot with the *Hongshanosaurus* taphomorphotype. IVPP V12617 plots as slightly negative on PC1 suggesting that it is neither strongly dorsoventrally crushed on the rostrum or mediolaterally crushed. It plots on opposite ends of the morphospace from IVPP V12704 in both PC2 and PC3. IVPP V12617 plots with a negative PC2 together with ZMNH M8138 because of the tall rostral aspect of the skull relative to the caudal aspect. IVPP V12617 plots on the positive PC3 axis due to having a tall rostrum and compressed caudal aspect. Though the positive PC3 axis preferentially shows crushing on the right side of the skull, IVPP V12617 is reasonably symmetric.

### (b) Application of morphometrics in dinosaurian paleontology

Morphometrics has been widely used in biology and paleontology in order to understand sexual dimorphism, individual variation, and interspecific variation. In spite of the application of traditional morphometrics to dinosaurs early in the development of the field of morphometrics [17], [19], [29], [32], [51], 2D geometric morphometrics has only recently been applied to dinosaur paleobiology in order quantitatively assess variation [20], [23], [28], [29]. In spite of the obvious advantages of 2D geometric morphometrics to traditional morphometrics in the replication of results, removal of observer bias, and higher statistical power [52]–[55], it is most useful when applied to objects that have a reasonably flattened surface (e.g. footprints [56]; trilobites [57]; leaves [58]), such that depth between landmarks does not create a source of error. It is not well suited for studies on complex three-dimensional shapes such as skulls [33]. For these objects, 3D geometric morphometrics is a logical extension, as this study demonstrates.

3D geometric morphometrics cannot be directly applied to cladistic analyses, since quantitative characters tend not to allow delineation of taxa as accurately as qualitative characters [33]. This is partially due to the taphonomic component of many character states [59]. Therefore, when examining interspecific variation using geometric morphometrics it is necessary also to analyze qualitative characters separating taxa. Combining these two approaches creates a powerful analytical tool for determining variation among closely related taxa.

Studies on extant and recently extinct forms do not have the issue of taphonomic distortion. However, extinct forms and especially forms from deep time such as dinosaurs often have a substantial degree of taphonomic distortion [60]–[63]. In the case of this study as well as most studies employing geometric morphometrics on dinosaurs, taphonomic distortion is likely to be a large factor affecting variation. Therefore, it is paramount to understand this important limitation when using this technique. This study also calls into question the usefulness of proportional characters in spite of their quantitative nature. *P. major* was considered to have a distinctive shape of its lateral temporal fenestra compared to other species of *Psittacosaurus* [10]. However, variation in this shape and proportion vary on different sides of the same skull (as in ZMNH M8137). The wide variability of these forms is clear in *Psittacosaurus* across a spectrum of skulls in a large sample size. However, such variability is not clear in species that are based on a single specimen. Even in extant animals, it has been shown that qualitative characters are more effective at distinguishing taxonomic groups [33]. Therefore, the use of proportional characteristics in cladistic analyses should only be done in samples where taphonomic deformation is not a factor.

Arbour and Currie [63] recently presented a method for retrodeforming ankylosaur skulls using finite element analysis. Though this technique was capable of reinflating ankylosaur skulls to their presumptive original shapes, the large amount of deformation in some psittacosaurid skulls suggests that this technique would only work for moderately deformed specimens. Additional studies on retrodeformation suggest that although many retrodeforming techniques create a greater degree of bilateral symmetry in samples, they do not reinflate objects to their original proportions [59], [62]. Though we interpret ZMNH M8137 as reasonably undistorted based on its location in the morphospace, it still displays small-scale taphonomic deformation. It is not possible to know for sure that this skull shape was definitively the shape that *P. lujiatunensis* had in life. Large amounts of taphonomic distortion were present in some samples, such as IVPP V12704 and DMNH D2590, which were only vaguely reminiscent of the original skull shape. When deformation is extreme, Arbour and Currie's [63] technique would be less effective since there is a larger amount of uncertainty in the reconstruction. Therefore, we did not attempt to apply retrodeforming techniques to our sample, but instead quantified the degree of taphonomic variation in the sample.

In spite of the difficulty of using 3D geometric morphometrics alone to understand interspecific variation, it can be effectively used to determine the amount of shape variation in a given sample. Using this technique it is possible to examine whether species with widely disparate shapes such as *P. sibiricus* [49] or other basal ceratopsians such as *Yinlong* [64], *Archaeoceratops* [65], [66], and *Auroraceratops* [67] plot within the same confines of the Lujiatun psittacosaurid cluster or outside of that cluster thereby adding intergeneric variation to the currently defined morphospace. It is clear that at some point intergeneric variation will swamp taphonomic distortion as species become more and more disparate in shape. Quantifying the degree to which intergeneric variation swamps taphonomic variation will be an important future study before further applying this technique further.

## Conclusions

It is evident from a reanalysis of characters and placement within a 3D geometric morphometric morphospace that the three Lujiatun psittacosaurids, *P. lujiatunensis*, *P. major*, and *Hongshanosaurus* are synonymous in spite of demonstrating seemingly distinctive shapes. Each nominal species represents a unique taphomorphotype (Figure 5). 3D geometric morphometrics has been used as a powerful tool for determining interspecific variation in shape in extant samples, but defines a single grouping within a taphomorphospace in this sample due to the high variability in the degree of taphonomic distortion of the studied skulls. The radical differences in shape among the conspecific sample of Lujiatun psittacosaurids demonstrate the potential for dramatic differences in intraspecific shape in extinct animals from deep time. This has implications for a high degree of shape variation in other dinosaurian samples as well, likely also due to taphonomic distortion. Based on these results, it is not likely that 3D geometric morphometrics will be capable of distinguishing taphonomically distorted specimens on the species level without employing retrodeformational techniques. This study represents the first attempt at quantification of variation in dinosaurs using 3D geometric morphometrics. Given the tremendous potential of this method, there are an endless number of applications to dinosaurian paleobiology. Future studies will determine when skull shapes in *Psittacosaurus* are different enough to stand in a morphospace as distinct species without being swamped by taphonomic distortion.

## Supporting Information

File S1 **Supplementary datasets, figures, tables, and multimedia.**
(PDF)

Multimedia S1 **ZMNH M8137.** A 3D model of ZMNH M8137 is included to demonstrate the scans for each psittacosaur specimen. ZMNH M8137 plotted closest to the consensus shape in each PC plot and likely represents a reasonably undistorted Lujiatun psittacosaur form. This file is an .obj file and can be visualized in MeshLab_TM_, which can be downloaded for free (MeshLab, Visual Computing Lab - ISTI - CNR http://meshlab.sourceforge.net/).
(ZIP)
