# Supplementary material for: Lujiatun Psittacosaurids: Understanding Individual and Taphonomic Variation Using 3D Geometric Morphometrics
Source: PLoS One. 2013 Aug 9;8(8):e69265. doi: 10.1371/journal.pone.0069265 (PMC3739782; doi:10.1371/journal.pone.0069265)
Supplement: File S1 — Supplementary datasets, figures, tables, and multimedia. (PDF) [file pone.0069265.s001.pdf]

Hedrick and Dodson: **Lujiatun Psittacosaurids: Understanding individual and taphonomic variation using 3D geometric morphometrics**

Supplemental Information:

25-Specimen dataset (without D3075-1, D3075-3, IVPP V12704)

The PCA was rerun not including any of the juvenile skulls (IVPP V12704, DMNH D3075-1, DMNH D3075-3) in order to test clusters in the morphospace with only adult individuals. All specimens clustered together in all PC axes demonstrating that allometry was not affecting the clustering seen in the 28-specimen dataset. The first four principal components are used for the 25-specimen dataset. Together they account for 62.3% of the total variance. For the 25-specimen dataset, the 95% confidence interval of the mean for PC1 is -0.172≤ μ ≥0.172. The confidence interval for PC2 is -0.142≤ μ ≥0.142, PC3 is -0.103≤ μ ≥0.103, and PC4 is -0.095≤ μ ≥0.095. The first principal component is composed of 28.2% of the total variance. The second principal component is composed of 15.4% of the total variance. The third principal component is composed of 9.85% of the total variance. The fourth principal component is composed of 8.85% of the total variance. Principal components 5–25 are individually composed of less than 7% of the total variance.

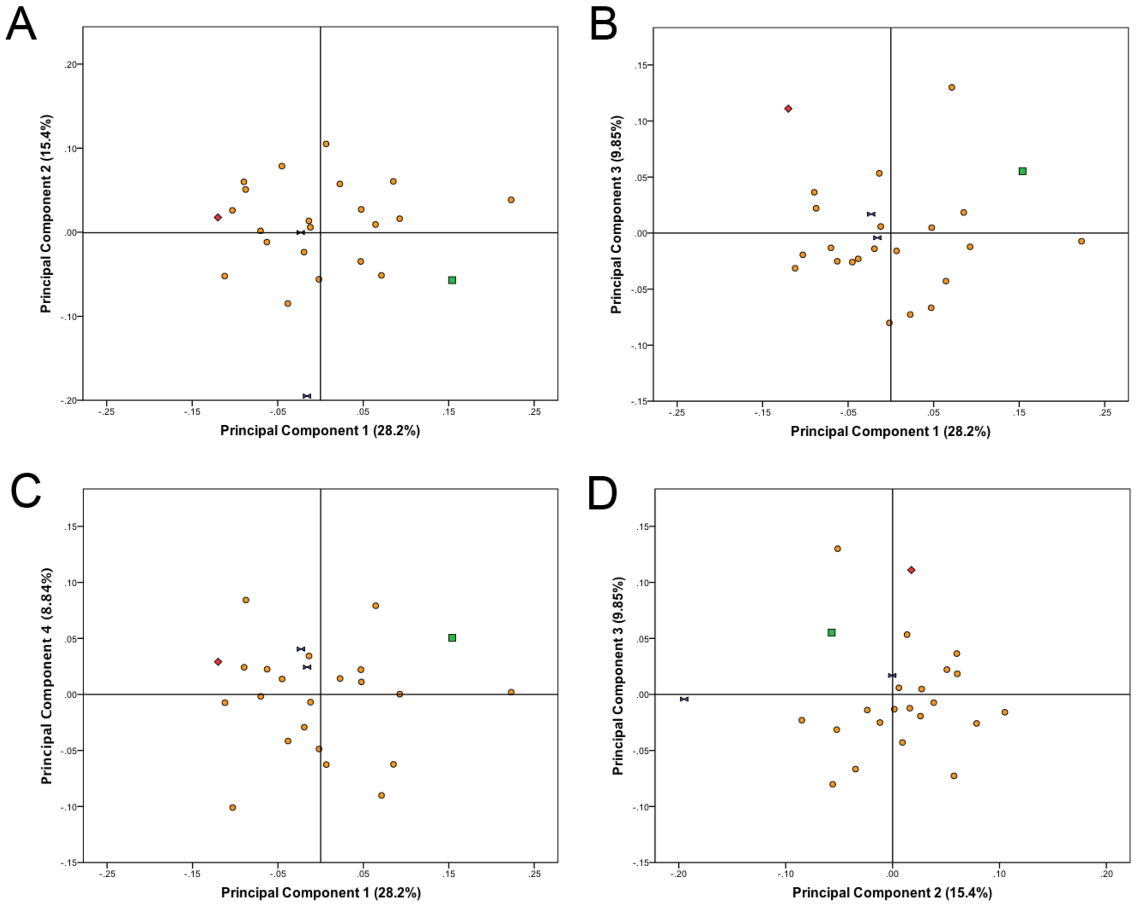

Figure S1: **PCA for 25-specimen dataset:** (A) PC1 x PC2, (B) PC1 x PC3, (C) PC1 x PC4, (D) PC2 x PC3. Specimens D3075-1, D3075-3 and IVPP V12704 were excluded in order to determine the organization of the adult-only morphospace.

26-Specimen dataset (without D3075-1, D3075-3, including IVPP V12704)

The PCA was rerun not using D3075-1 or D3075-3 given that the juvenile skulls were occupying a different morphospace from the larger skulls. Though IVPP V12704 was also a juvenile, it was not discounted from these PCAs because of its status as the holotype of *Hongshanosaurus houi*. In general, this caused the grouping to be more closely aligned with the 95% confidence ellipses with the exception of IVPP V12704. The first four principal components are used for the 26-specimen dataset. Together, they comprise 63.8% of the total variance. The confidence interval for each PC is determined by the mean based on all 26 specimens and is displayed graphically in the confidence ellipse. The confidence interval for PC1 is $-0.172 \leq \mu \geq 0.172$. The confidence interval for PC2 is $-0.142 \leq \mu \geq 0.142$. The confidence interval for PC3 is $-0.103 \leq \mu \geq 0.103$. The confidence interval for PC4 is $-0.095 \leq \mu \geq 0.095$. The first principal component is composed of 27.1% of the total variance. Principal component 2 is composed of 18.5% of the total variance. Principal component 3 is composed of 9.8% of the total variance. Principal component 4 is composed of 8.36% of the total variance.

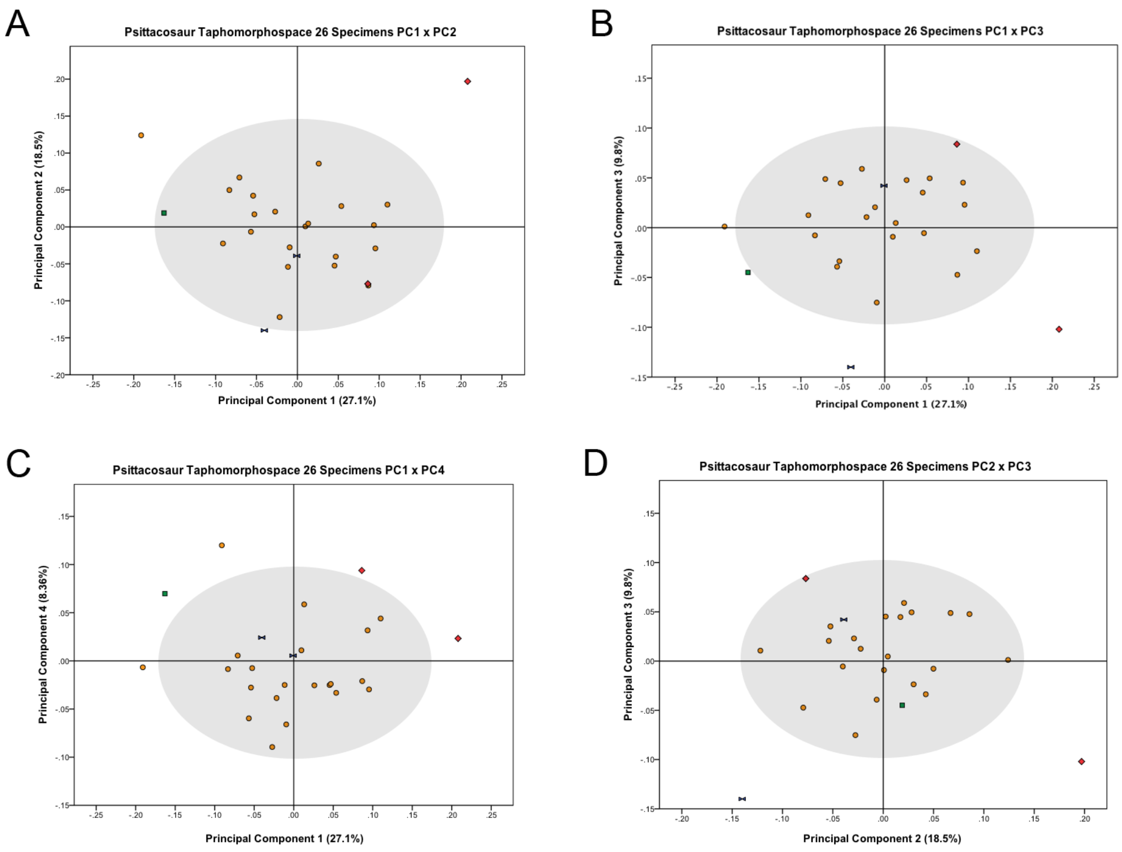

Figure S2: **PCA for 26-specimen dataset:** (A) PC1 x PC2, (B) PC1 x PC3, (C) PC1 x PC4, (D) PC2 x PC3. Specimens D3075-1 and D3075-3 were excluded and IVPP V12704 was retained.

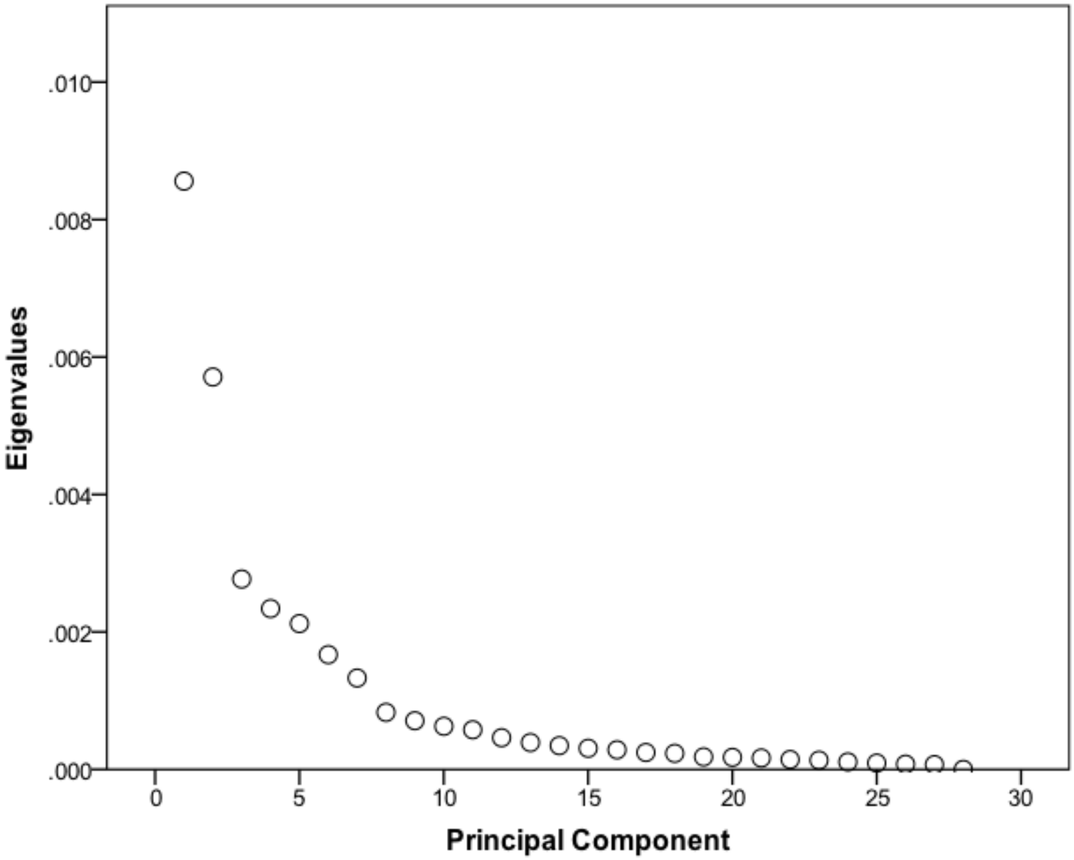

Figure S3: **Scree plot**: This is the scree plot for all 28 principal component generated from the 28-specimen dataset.

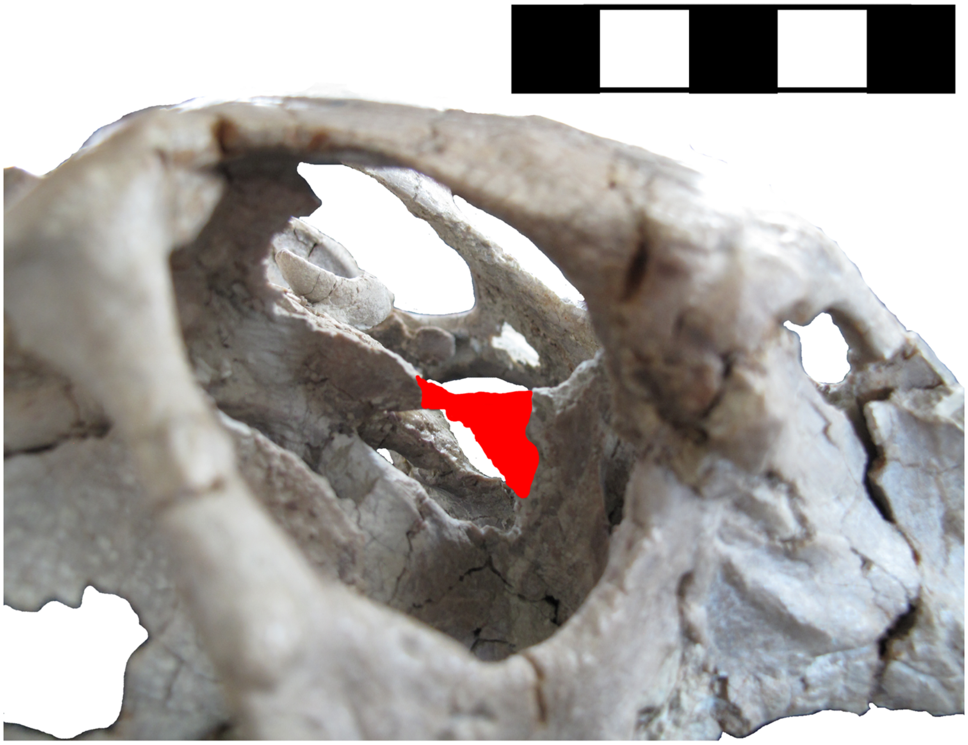

Figure S4: ***Hongshanosaurus* (IVPP V12617):** The interior of the skull is connected by plaster (red) showing that the back of the skull was taphonomically distorted such that the back of the skull was angled relative to the front of the skull. This explains the elongated rostrum. Scale =50mm.

Table S1: **28-specimen dataset PC coords:** The 28-specimen dataset principal component coordinates and ln(centroid size). Only the first four principal components were used in the analysis and a total of 28 principal components were found to account for 100% of the total variance. Only the first 12 principal components are presented here accounting for 90% of the total variance.

28-Specimen Datasheet

Specimen	PC1	PC2	PC3	PC4	PC5	PC6
D1882	-3.88E-02	-1.12E-01	-4.29E-02	-1.53E-02	1.70E-03	4.06E-02
D2156	-4.69E-02	-8.48E-02	8.71E-03	2.52E-02	-1.15E-01	1.59E-02
D2581	4.78E-02	-5.16E-02	1.56E-02	-1.62E-02	-3.28E-02	-8.42E-03
D2582	-4.82E-02	6.13E-02	2.82E-02	-2.67E-03	-7.22E-02	3.74E-02
D2583	3.50E-02	-3.15E-02	-7.85E-02	-1.30E-02	-6.40E-02	6.05E-03
D2585	5.62E-02	7.95E-02	3.98E-02	3.44E-02	-7.06E-02	-1.93E-02
D2586	-8.90E-03	-7.00E-02	3.00E-02	-3.26E-02	-7.88E-03	-4.45E-03
D2587	4.47E-02	3.99E-02	4.12E-02	-2.92E-02	1.43E-02	3.95E-03
D2588	7.28E-02	6.91E-02	-1.53E-02	8.49E-03	-2.01E-02	-3.16E-02
D2589	-7.43E-02	-4.04E-02	4.80E-02	1.61E-02	2.02E-02	-1.04E-02
D2590	1.34E-01	1.87E-01	-1.11E-02	-2.71E-03	-8.30E-03	-1.13E-03
D2591	6.15E-02	1.44E-02	-4.89E-02	-5.85E-02	6.59E-03	9.70E-03
D2592	1.67E-02	3.42E-02	3.44E-02	-1.04E-01	-1.75E-02	-8.83E-03
D2593	1.17E-02	-1.69E-02	-1.21E-02	2.95E-02	-2.14E-02	-2.71E-02
D2595	8.40E-02	-1.08E-01	9.80E-03	-3.07E-02	-3.47E-02	-7.13E-03
D2596	-4.48E-02	-3.63E-03	3.90E-02	-2.12E-02	-2.80E-02	-6.00E-02
D2597	2.90E-03	-9.75E-03	1.51E-02	4.85E-02	3.51E-02	-2.72E-02
D2598	4.22E-02	5.50E-02	-4.13E-02	-3.41E-02	3.99E-02	-5.63E-02
D2600	-2.23E-02	-5.48E-02	-4.69E-03	-3.84E-02	2.38E-02	2.15E-02
D30751	-1.70E-01	1.15E-01	7.23E-02	-2.53E-02	6.02E-02	7.32E-02
D30753	-1.61E-01	4.53E-02	-5.76E-02	-7.02E-02	2.44E-02	3.01E-02
D3419	-9.47E-02	-2.83E-02	-1.27E-02	4.63E-02	4.81E-02	-8.22E-02
DL0013	1.05E-01	1.52E-02	4.95E-02	1.16E-01	3.45E-03	1.13E-01
IVPP V12617	-3.08E-02	-1.09E-01	1.06E-01	5.72E-02	5.00E-02	-4.09E-02
LHPV1	1.57E-01	7.51E-02	-3.44E-02	5.15E-02	6.21E-02	-2.68E-02
ZMNH M8137	1.37E-02	-2.63E-02	4.81E-02	-4.22E-02	5.94E-02	2.09E-02
ZMNH M8138	1.02E-01	-1.17E-01	-1.19E-01	1.42E-02	6.58E-02	5.03E-02
IVPPV12704	-2.47E-01	7.21E-02	-1.07E-01	8.88E-02	-2.21E-02	-1.09E-02

PC7	PC8	PC9	PC10	PC11	PC12	Ln(Centroid Size)
7.95E-03	1.51E-02	-2.94E-02	-5.76E-02	5.50E-02	1.13E-02	6.051575795
7.67E-02	3.13E-02	3.50E-02	-3.02E-02	-4.55E-02	-1.32E-02	5.771704436
-7.91E-03	2.02E-03	-1.15E-02	-7.20E-03	4.64E-03	2.31E-04	5.915541968
2.46E-02	-6.99E-02	8.39E-03	-5.23E-03	1.74E-02	1.70E-02	5.265097228
-1.09E-01	-1.22E-02	4.02E-02	-4.48E-03	-4.71E-03	-2.82E-02	5.412674457
-4.06E-02	-2.16E-02	-6.32E-02	-1.25E-03	-4.89E-03	1.14E-02	5.772562751
1.86E-02	1.61E-02	1.16E-02	3.31E-02	-3.51E-03	-9.74E-03	5.86007845
1.87E-03	-2.05E-04	-1.39E-02	2.80E-02	-9.12E-03	7.98E-03	6.014952747
-2.03E-02	-6.43E-03	1.08E-02	-1.86E-03	4.16E-04	-2.31E-03	5.886083012
4.50E-02	2.41E-03	5.87E-03	-4.73E-03	1.13E-02	-1.70E-02	5.901501267
4.71E-02	5.18E-02	-3.79E-02	-7.12E-03	1.59E-03	-1.18E-02	5.6487102
1.90E-02	4.22E-02	4.53E-03	1.19E-02	3.80E-02	2.73E-02	6.034633773
1.78E-02	-7.21E-03	2.81E-02	2.80E-03	1.14E-02	1.37E-03	6.141975564
7.45E-03	-2.04E-02	7.12E-03	-2.16E-02	4.01E-03	-2.34E-03	5.739504485
-3.23E-02	3.90E-02	-4.69E-02	2.40E-02	-2.15E-02	-2.75E-02	5.873502823
-3.50E-02	-1.20E-02	8.68E-03	1.08E-03	-6.20E-03	6.16E-02	5.85155821
2.49E-02	-3.18E-03	1.34E-03	-3.63E-03	-3.52E-02	3.00E-02	5.653011258
-8.08E-03	3.37E-03	4.12E-02	6.79E-03	2.19E-02	-2.49E-02	5.908835599
6.60E-03	2.09E-03	1.30E-02	1.36E-02	2.54E-02	1.58E-02	6.084254994
-6.38E-02	4.39E-02	1.05E-02	-4.65E-02	-2.64E-02	2.31E-03	5.180570763
2.36E-02	-6.37E-02	-4.49E-02	1.89E-03	-1.47E-02	-3.86E-02	5.280425719
-2.89E-02	1.87E-02	-6.02E-03	-2.50E-02	-6.86E-03	-5.64E-03	5.820858115
-2.22E-02	-1.28E-03	2.23E-02	2.65E-02	2.37E-02	-7.04E-03	5.943697253
-3.76E-03	-1.68E-02	-1.80E-02	7.39E-03	3.34E-02	-2.41E-02	5.980623583
3.24E-02	-3.09E-02	2.51E-02	-3.32E-02	-8.28E-03	-1.23E-02	6.134333856
7.30E-03	-1.17E-02	1.75E-02	3.78E-02	-3.56E-02	1.71E-03	6.203464179
-1.19E-04	-1.74E-02	-1.95E-02	-1.06E-03	-3.67E-02	2.96E-02	6.148098058
1.05E-02	2.69E-02	7.16E-06	5.58E-02	1.10E-02	7.13E-03	4.550260059

Centroid Size
424.782
321.085
370.755
193.465
224.23
321.36
350.752
409.506
359.992
365.586
283.925
417.646
464.971
310.91
355.492
347.776
285.149
368.277
438.893
177.784
196.453
337.261
381.342
395.687
461.432
494.459
467.827
94.657

Table S2: **Error test data:** The error test with Euclidean distances of the first four principal components of each specimen. Euclidean distances were plotted in Figure 2 and shown to have the error sample (LHPV1) not overlap with the other specimens.

	Specimens	PC1	PC2	PC3	PC4
1	D1882	-1.25E-01	8.20E-02	-5.61E-02	-2.52E-02
2	D2156	-1.36E-01	6.44E-02	1.58E-02	-2.59E-02
3	D2581	-4.19E-02	7.94E-02	3.36E-02	-1.48E-02
4	D2582	-8.18E-02	-6.11E-02	6.66E-02	-2.35E-02
5	D2583	-4.51E-02	5.54E-02	-4.77E-03	-1.04E-01
6	D2585	5.70E-03	-2.33E-02	9.98E-02	-1.72E-02
7	D2586	-9.50E-02	6.68E-02	1.88E-02	9.18E-03
8	D2587	-5.22E-03	-2.01E-03	6.07E-02	1.01E-02
9	D2588	3.63E-02	-1.70E-02	3.67E-02	-3.42E-02
10	D2589	-1.26E-01	3.90E-03	-6.84E-03	6.33E-02
11	D2590	1.30E-01	-8.61E-02	8.90E-02	-5.29E-02
12	D2591	7.52E-04	2.60E-02	1.07E-02	-7.03E-02
13	D2592	-3.89E-02	-6.49E-03	7.85E-02	-3.40E-02
14	D2593	-4.40E-02	2.49E-02	-8.23E-03	-5.07E-03
15	D2595	-4.59E-02	1.50E-01	3.90E-02	-3.15E-02
16	D2596	-9.89E-02	-7.31E-03	3.74E-02	9.67E-03
17	D2597	-3.95E-02	1.02E-02	-2.03E-02	5.06E-02
18	D2598	1.73E-02	-2.60E-02	-1.07E-02	-3.17E-02
19	D2600	-9.00E-02	4.21E-02	-1.55E-02	-3.04E-03
20	D30751	-1.54E-01	-1.71E-01	4.94E-02	5.86E-02
21	D30753	-1.58E-01	-1.15E-01	-4.92E-02	-4.82E-02
22	D3419	-1.24E-01	-2.38E-02	-7.00E-02	4.84E-02
23	DL0013	3.73E-02	4.79E-02	5.35E-02	5.31E-02
24	IVPP V12617	-1.15E-01	8.38E-02	1.44E-04	1.39E-01
25	LHPV1	1.52E-01	-5.84E-04	-2.83E-02	1.77E-02
26	LHPV1-1	1.42E-01	-2.19E-02	-2.57E-02	1.40E-02
27	LHPV1-2	1.44E-01	-1.61E-02	-1.66E-02	1.03E-02
28	LHPV1-3	1.44E-01	-1.68E-02	-1.66E-02	6.41E-03
29	LHPV1-4	1.45E-01	-1.49E-02	-1.54E-02	7.18E-03
30	LHPV1-5	1.43E-01	-1.84E-02	-2.31E-02	1.24E-02
31	LHPV1-6	1.44E-01	-2.05E-02	-2.64E-02	9.59E-03
32	LHPV1-7	1.43E-01	-2.28E-02	-2.46E-02	1.13E-02
33	LHPV1-8	1.45E-01	-2.22E-02	-2.66E-02	1.30E-02
34	LHPV1-9	1.43E-01	-2.03E-02	-2.28E-02	1.33E-02
35	LHPV1-10	1.45E-01	-2.19E-02	-2.37E-02	1.30E-02
36	ZMNH M8137	-4.88E-02	3.54E-02	2.14E-02	4.40E-02
37	ZMNH M8138	1.03E-02	1.44E-01	-1.12E-01	-5.02E-02
38	IVPPV12704	-2.16E-01	-1.82E-01	-1.08E-01	-4.17E-02

Mean of Error
PC1
1.45E-01

			EuclideanDistance (PCs)
			0.291716417
			0.297261636
			0.219295535
			0.2496285
			0.234582135
			0.187479829
			0.25744376
			0.172163243
			0.131718332
			0.276744121
			0.146680559
			0.174447895
			0.214729612
			0.194593188
			0.264791052
			0.250985817
			0.190216716
			0.135208931
			0.242639219
			0.346391587
			0.324430873
			0.275206866
			0.15280955
PC2	PC3	PC4	0.307305687
-1.79E-02	-2.27E-02	1.17E-02	0.020533376
			0.006108358
			0.006520867
			0.00813718
			0.009074991
			0.001843574
			0.005018097
			0.005528136
			0.00600471
			0.003331301
			0.0044024
			0.207870908
			0.236678878
			0.408727642

Table S3: **Psittacosaur skull and femur lengths:** The skull and femur ratios for a number of specimens. Each side of the body is shown in the table as there is often some discrepancy. This was used to assess the relative skull size of *P. major*.

Specimen Number	Side	Skull	Femur	Skull - Femur Ratio
D2594	Right	127	121.9	1.041837572
	Left	127	121.4	1.046128501
D2595	Right	132.7	130.7	1.015302219
	Left	132.7	131.4	1.009893455
D2591	Right	183.8	183.2	1.003275109
	Left	183.8	170.9	1.075482738
D2588	Right	155.4		
	Left	155.4	129.7	1.198149576
D2586	Right	133.8	137.4	0.973799127
	Left	133.8	134.3	0.996276992
D2587	Right	168	164.6	1.020656136
	Left	168	166.3	1.010222489
D2585	Right	131.9	124.8	1.056891026
	Left	131.9	129.2	1.020897833
D3419	Right	130.8	126.9	1.030732861
	Left	130.8		
D2593	Right	115.7	119.2	0.970637584
	Left	115.7	119.2	0.970637584
D2590	Right	112.2	104.8	1.070610687
	Left	112.2	107.1	1.047619048
D2584	Right	136.7	108.4	1.261070111
	Left	136.7	108.9	1.255280073
D2583	Right	82.6	90.7	0.910694598
	Left	82.6	91.6	0.901746725
D2582	Right	86.1	71.2	1.209269663
	Left	86.1	70	1.23
D2581	Right	143.1	130.3	1.098234843
	Left	143.1	131.2	1.09070122
D2589	Right	142.5	122.4	1.164215686
	Left	142.5	130.6	1.091117917
D1882	Right	176.6	130.9	1.349121467
	Left	176.6	141	1.25248227
D1883	Right		112	
	Left		113.5	
D2602-1	Right	91.2		
	Left	91.2		
D2602-2	Right	61	71.6	0.851955307
	Left	61		
D2602-3	Right	95.4	76.2	1.251968504
	Left	95.4	74.1	1.287449393
D2602-4	Right	54.1		
	Left	54.1		
D2600 (B)	Right	178.3	179.8	0.991657397
	Left	178.3	179.4	0.99386845
D2598 (A)	Right	148.2	133.1	1.113448535

	Left	148.2	130	1.14
D2601-1	Right	48.9		
	Left	48.9		
D2601-2	Right	34.6	46.7	0.740899358
	Left	34.6	44.5	0.77752809
D2601-3	Right	46	36.3	1.267217631
	Left	46	33.2	1.385542169
D2601-4	Right	56.2		
	Left	56.2	50.8	1.106299213
D2601-5	Right	46.4		
	Left	46.4		
D3075-1	Right	65.8		
	Left	65.8		
D3075-2	Right	67	76.2	0.879265092
	Left	67	75.5	0.887417219
D2599 (E)	Right		86.3	
	Left		88.6	
ZMNH M8137	Right	185.3	186.9	0.991439272
	Left	185.3	177.6	1.043355856
ZMNH M8138	Right	206.4	200.8	1.027888446
	Left	206.4	196.4	1.050916497
IVPP V738 (sinensis	Right	113.3	97.4	1.163244353
	Left	113.3	97.5	1.162051282
IVPP V740	Right		96.7	
	Left		96.7	
IVPP V7398	Right			
	Left			
IVPP 14341-1		58.6	53	1.105660377
IVPP 14341-2		60.9		
IVPP 14341-3		73.1	68.1	1.073421439
IVPP 14341-4		68.4	62.1	1.101449275
IVPP 14341-5		91.2	77.7	1.173745174
LHPV1	Right	193.2	171.6	1.125874126
	Left	193.2	168.5	1.146587537
LHPV2	Right	105.5		
	Left	105.5	130	0.811538462

Table S4: **25-specimen dataset PC coords:** The 25-specimen dataset principal component coordinates for the first four principal components. This dataset eliminated small (juvenile) specimens from the analysis to assess any potential grouping in only the adult specimens. (see Figure S1 for groupings).

25-Specimen Datasheet

Specimen	PC1	PC2	PC3	PC4	Centroid Size	Ln Centroid Size
D1882	-1.12E-01	-5.23E-02	-3.14E-02	-7.38E-03	424.7818722	6.051575795
D2156	-1.03E-01	2.60E-02	-1.94E-02	-1.01E-01	321.0845347	5.771704436
D2581	-1.92E-02	-2.37E-02	-1.40E-02	-2.93E-02	370.7551855	5.915541968
D2582	6.63E-03	1.05E-01	-1.59E-02	-6.26E-02	193.465118	5.265097228
D2583	-1.82E-03	-5.60E-02	-8.02E-02	-4.87E-02	224.2304812	5.412674457
D2585	8.52E-02	6.05E-02	1.84E-02	-6.24E-02	321.3602445	5.772562751
D2586	-7.01E-02	1.60E-03	-1.32E-02	-1.73E-03	350.7516593	5.86007845
D2587	4.77E-02	2.72E-02	4.82E-03	1.11E-02	409.506488	6.014952747
D2588	9.26E-02	1.61E-02	-1.23E-02	2.26E-04	359.992433	5.886083012
D2589	-8.96E-02	6.00E-02	3.64E-02	2.42E-02	365.5858981	5.901501267
D2590	2.23E-01	3.85E-02	-7.31E-03	2.01E-03	283.925023	5.6487102
D2591	4.71E-02	-3.47E-02	-6.66E-02	2.20E-02	417.6458285	6.034633773
D2592	2.27E-02	5.74E-02	-7.27E-02	1.42E-02	464.9712443	6.141975564
D2593	-1.19E-02	5.87E-03	5.87E-03	-6.92E-03	310.910312	5.739504485
D2595	-3.82E-02	-8.49E-02	-2.30E-02	-4.16E-02	355.4920274	5.873502823
D2596	-4.51E-02	7.86E-02	-2.59E-02	1.38E-02	347.7758662	5.85155821
D2597	-1.36E-02	1.35E-02	5.33E-02	3.43E-02	285.148831	5.653011258
D2598	6.44E-02	9.18E-03	-4.29E-02	7.91E-02	368.2770833	5.908835599
D2600	-6.27E-02	-1.18E-02	-2.51E-02	2.25E-02	438.892713	6.084254994
D3419	-8.75E-02	5.08E-02	2.21E-02	8.42E-02	337.2613384	5.820858115
DL0013	7.13E-02	-5.14E-02	1.30E-01	-9.01E-02	381.3422453	5.943697253
IVPP V12617	-1.20E-01	1.76E-02	1.11E-01	2.91E-02	395.6870348	5.980623583
LHPV1	1.54E-01	-5.70E-02	5.52E-02	5.05E-02	461.4316119	6.134333856
ZMNH M8137	-2.31E-02	-3.53E-04	1.69E-02	4.05E-02	494.4589719	6.203464179
ZMNH M8138	-1.58E-02	-1.95E-01	-4.17E-03	2.43E-02	467.8267606	6.148098058

Table S5: **Full PCs and Eigenvalues for the 28-specimen dataset.**

Principal Component Number	Eigenvalues	Proportion of Total Variance	Cumulative Variance
PC1	8.56E-03	2.79E-01	2.79E-01
PC2	5.71E-03	1.86E-01	4.65E-01
PC3	2.77E-03	9.04E-02	5.56E-01
PC4	2.34E-03	7.64E-02	6.32E-01
PC5	2.12E-03	6.92E-02	7.01E-01
PC6	1.67E-03	5.45E-02	7.56E-01
PC7	1.33E-03	4.35E-02	7.99E-01
PC8	8.29E-04	2.70E-02	8.26E-01
PC9	7.09E-04	2.31E-02	8.49E-01
PC10	6.27E-04	2.04E-02	8.70E-01
PC11	5.77E-04	1.88E-02	8.89E-01
PC12	4.60E-04	1.50E-02	9.04E-01
PC13	3.90E-04	1.27E-02	9.16E-01
PC14	3.46E-04	1.13E-02	9.28E-01
PC15	3.07E-04	1.00E-02	9.38E-01
PC16	2.86E-04	9.33E-03	9.47E-01
PC17	2.46E-04	8.04E-03	9.55E-01
PC18	2.33E-04	7.59E-03	9.63E-01
PC19	1.82E-04	5.93E-03	9.69E-01
PC20	1.74E-04	5.67E-03	9.74E-01
PC21	1.65E-04	5.37E-03	9.80E-01
PC22	1.45E-04	4.71E-03	9.84E-01
PC23	1.33E-04	4.33E-03	9.89E-01
PC24	1.09E-04	3.56E-03	9.92E-01
PC25	9.13E-05	2.98E-03	9.95E-01
PC26	7.49E-05	2.44E-03	9.98E-01
PC27	6.98E-05	2.28E-03	1.00E+00
PC28	6.00E-12	1.96E-10	1.00E+00

Multimedia S1: **ZMNH M8137:** A 3D model of ZMNH M8137 is included to demonstrate the scans for each psittacosaur specimen. ZMNH M8137 plotted closest to the consensus shape in each PC plot and likely represents a reasonably undistorted Lujiatun psittacosaur form. This file is an .obj file and can be visualized in MeshLab^{TM}, which can be downloaded for free (MeshLab, Visual Computing Lab - ISTI - CNR http://meshlab.sourceforge.net/)
